# Enhancing Railway Earthquake Early Warning Systems with a Low Computational Cost STA/LTA-Based S-Wave Detection Method

**DOI:** 10.3390/s24237452

**Published:** 2024-11-22

**Authors:** Satoshi Katakami, Naoyasu Iwata

**Affiliations:** Railway Technical Research Institute, 2-8-38, Hikari-cho, Kokubunji 185-8540, Tokyo, Japan

**Keywords:** real-time algorithm, earthquake early warning, STA/LTA, S-wave picking, K-net

## Abstract

To enhance real-time S-wave detection in the railway earthquake early warning (EEW) system, we improved the existing short-term average/long-term average (STA/LTA) algorithm. This enhancement focused on developing a more robust and computationally efficient method. Specifically, we introduced noise reflecting P-wave amplitude information before the P-wave to better distinguish between P- and S-waves. By applying this modified STA/LTA method, we achieved a significant improvement in S-wave detection accuracy. For seismic waveforms from stations located within 100 km of the epicenter of each earthquake, with magnitude of M5.5–6.5 and depths ≤ 100 km, the detection accuracy within 1.5 s of the correct time (manual picking) was 81.0%, compared to the 49.0% accuracy of the currently operational railway EEW system. Importantly, despite the improved accuracy, the computational cost of the new method remains comparable to the existing system, allowing for easy integration into the operational EEW system. This development is crucial for preventing false alarms, especially moderate earthquakes (~M6) because issuing warn-ings in unnecessary areas can have a significant social impact. Future plans involve implementing this method into the current system to further improve early warning capabilities and minimize false alarms.

## 1. Introduction

The earthquake early warning (EEW) system is a highly effective method for mitigating earthquake hazards. Typically, the EEW system provides crucial information such as magnitude and location (hypocenter or epicenter) within seconds after detecting the initial P-wave at the first station. In Japan, practical EEW systems have been under development for nearly 30 years. Ref. [1] introduced the Urgent Earthquake Detection and Alarm System (UrEDAS) to safely stop the Shinkansen (bullet train) during earthquakes. The railway EEW systems rapidly analyze seismic waveforms acquired from stations in close proximity to the hypocenter and estimate critical parameters, such as hypocentral distance, back azimuth, and magnitude [2,3,4,5,6,7,8]. The swift and accurate determination of magnitudes is a crucial element of the EEW system. Magnitudes are usually calculated by using the epicentral distance estimated through the aforementioned process, as well as the maximum amplitude recorded at each station. To facilitate the timely estimation of magnitude immediately after an earthquake detection, the EEW process employs two types of magnitude calculation formulas, one for the P-wave phase (P-wave magnitude) and the other after the arrival of the S-wave (all-phase magnitude). The process dynamically switches between the two formulas depending on the estimated arrival time of the S-wave at each observation point. Hence, the accurate real-time determination of the S-wave arrival time is important for precise magnitude estimation. In a railway earthquake early warning (EEW) system, the real-time detection of S-waves involves monitoring the amplitude ratio of combined horizontal (two components) and vertical (one component) seismic waveforms over time [9]. When this ratio exceeds a certain threshold, it is interpreted as the arrival time of the S-wave. This method was adopted because of its extremely low computational cost and its fundamental similarity to the short-term average/long-term average (STA/LTA) [10,11] method used for P-wave detection. It was designed to operate on the machine specifications available at the time of its introduction, about 40 years ago. Although machine updates are gradually being implemented, the algorithm remains low in computational cost. The concept behind this method is that the amplitude of the horizontal component of the seismometer will increase upon the arrival of the S-wave. However, due to wave scattering, complex subsurface and fault structures, and converted waves from P-wave to S-wave, the threshold is often exceeded prematurely relative to the actual S-wave arrival time, leading to the underestimation of magnitude.

Real-time detection of S-waves is challenging due to their subtle features compared to the more pronounced differences between background noise and P-waves. The current method for detecting S-wave arrival times in railway EEW systems uses the amplitude ratio between horizontal and vertical seismic wave components, however, it is known that this method has a significant margin of error because it is difficult to identify these subtle features while P-waves are being recorded. Numerous techniques exist for real-time P-wave detection, but relatively few are available for S-waves. Traditional methods for picking P- and S-wave arrival times include the STA/LTA. Examples of S-wave picking methods incorporate various advanced techniques such as higher-order statistics, including kurtosis [12], an improved STA/LTA method using the envelope function [13], autoregressive prediction suitable for real-time processing [14], a non-parametric method for automatic determination of P-wave and S-wave arrival times [15], and combining singular value decomposition with the STA/LTA method [16]. The damped predominant period (Tpd) method, used for estimating both P- and S-wave arrival times, has demonstrated superior accuracy compared to STA/LTA pickers [17]. Autoregressive (AR) model fitting has also been employed for determining onset times of both P- and S-waves. Leonard and Kennett provide a comprehensive review of various AR models and methods for picking onset times using AR prediction. For P-wave onset time determination, the univariate approach [18] applied to the Z-component is preferred, while for S-wave onset time estimation, a multivariate AR model is recommended [19]. Deep learning techniques have recently made significant progress, offering new solutions for the fast and accurate pickup of P- and S-waves [20,21,22]. Despite these advancements, the development of optimized automated algorithms for S-wave detection remains an ongoing research topic. Robust automated procedures that include quality estimations can improve the consistency of automated S-phase picking by recognizing, downgrading, or rejecting uncertain and potentially incorrect arrival times, which can significantly influence hypocenter estimation.

In this study, we developed a simple method, by modifying the STA/LTA method, that robustly determines the arrival time of S-waves in real time, with low computational cost, to be incorporated into the railway EEW system. The STA/LTA algorithm is highly efficient for real-time processing owing to its low computational cost. However, in the implementation of STA/LTA in real-time S-wave detection, the utilization of P-wave amplitudes for calculating the LTA presents challenges, including the significant amplitude variation of P-waves in comparison with the background noise and the limited S-P time available for calculating LTA at stations situated near the hypocenter. Our developed method effectively stabilizes the STA/LTA fluctuations in the P-wave prior to the arrival of the S-wave while adhering to the basic STA/LTA algorithm, and clarifies the characteristic difference between P- and S-waves.

## 2. Data and Methods

We developed a real-time method for determining the arrival times of S-waves based on the principles of the STA/LTA method. First, the arrival time of the P-wave was determined using a conventional STA/LTA approach. Artificial noise, quantified in terms of the P-wave amplitude, was then introduced into the signal. Subsequently, STA/LTA was performed on the modified signal to determine the arrival time of the S-wave. Given that the proposed method involves the computation of STA/LTA twice, specifically for the P-wave and S-wave, hereafter, this method shall be referred to as the “Two-Step STA/LTA”.

The STA/LTA method has been used in many P-wave detection systems owing to its computational efficiency. For P-wave detection, the real-time calculation of ${STA}_{p}$ and ${LTA}_{p}$ is performed on the seismic waveform data, as follows:

(1) $${STA}_{p}\left(k\right)=\frac{1}{\alpha_{p}}\sum_{i=k-\alpha_{p}}^{k}\left|x_{i}\right|.$$

(2) $${LTA}_{p}\left(k\right)=\frac{1}{\beta_{p}}\sum_{i=k-\beta_{p}}^{k}\left|x_{i}\right|.$$

(3) $$\frac{{STA}_{p}}{{LTA}_{p}}>{th}_{p}.$$

where $x_{i}$ is the current sample of a data sequence, the parameters $\alpha_{p}$ and $\beta_{p}$ represent the length of time used to calculate the average value, and $\alpha_{p}\ll \beta_{p}$. The ${th}_{p}$ is the ${STA}_{p}/{LTA}_{p}$ threshold for P-wave detection. Specifically, the time *k*, at which Equation (3) is first satisfied, is identified as the P-wave arrival time *γ*. Next, the 90th percentile of the P-wave, denoted as *q*, is calculated from *γ* to *γ* + *δ* s as follows:

(4) $$q={percentile}_{90\%}\left\{x_{\gamma },x_{\gamma +1},x_{\gamma +2},\cdots ,x_{\gamma +\delta }\right\}.$$

Subsequently, the random noise (white noise) *N* with values ranging from 0 to 1, is multiplied by *q* to generate a noise waveform that incorporates the amplitude information of the P-wave from *γ* to *γ* + *δ*. The noise waveform, which is the product of *q* and *N*, is replaced with the original signal waveform $x_{i}$ from time *γ* + *δ* − *β* to time *γ* + *δ*. Therefore, waveform $y_{i}$ can be generated to detect the S-wave as follows:

(5) $$y_{i}=\left\{{q*N}_{\gamma +\delta -\beta },{q*N}_{\gamma +\delta -\beta +1},{q*N}_{\gamma +\delta -\beta +2},\cdots ,q*N_{\gamma +\delta },x_{\gamma +\delta +1},\cdots \right\}.$$

Although creating $y_{i}$ from a time earlier than *γ* + *δ* − *β* presents no issue, we generated only the essential $y_{i}$ required to compute the LTA to minimize the cost of the real-time computation. Next, the STA/LTA analysis is performed on the modified waveform $y_{i}$ to establish the ${STA}_{s}/{LTA}_{s}$ threshold, ${th}_{s}$ for detecting the S-wave, as follows:

(6) $${STA}_{s}\left(k\right)=\frac{1}{\alpha_{s}}\sum_{i=k-\alpha_{s}}^{k}\left|y_{i}\right|.$$

(7) $${LTA}_{s}\left(k\right)=\frac{1}{\beta_{s}}\sum_{i=k-\beta_{s}}^{k}\left|y_{i}\right|.$$

(8) $$\frac{{STA}_{s}}{{LTA}_{s}}>{th}_{s}.$$

where the parameters $\alpha_{s}$ and $\beta_{s}$ represent the length of time used to calculate the average value and $\alpha_{s}$≪$\beta_{s}$. $\alpha_{s}$ and $\beta_{s}$ can have an identical value to that of $\alpha_{p}$ and $\beta_{p}$. Finally, the time *k* at which Equation (8) is first satisfied is designated as the arrival time of the S-wave.

The *δ*, *q*, and $y_{i}$ values are updated if the ${STA}_{s}/{LTA}_{s}$ value does not surpass the threshold (${th}_{s}$). The amplitude of the P-wave may undergo gradual changes, leading to the *q* value becoming insufficient a few seconds after the P-wave arrival. Hence, we increment the *δ* value each second and update the *q* and $y_{i}$ until the ${STA}_{s}/{LTA}_{s}$ exceeds the ${th}_{s}$. First, we generate the *q* and $y_{i}$ based on the data between *γ* and *γ* + 2 s, and conduct ${STA}_{s}/{LTA}_{s}$. If the ${STA}_{s}/{LTA}_{s}$ values do not exceed the ${th}_{s}$ by *γ* + 3 s, we update the *q* and $y_{i}$ again based on the data between *γ* and *γ* + 3 s, followed by performing the ${STA}_{s}/{LTA}_{s}$ until *γ* + 4 s, using updated $y_{i}$. This iterative process continues until *γ* + 6 s, beyond which the *q* and $y_{i}$ are not updated.

The objective of this method is to stabilize the temporal fluctuations of the STA/LTA until the arrival of the S-waves. Directly following the onset of the P-wave, the computed LTA encompasses both the pre-P-wave noise and the P-wave itself. Consequently, the LTA value gradually escalates. In such instances, the STA/LTA requires time to stabilize, and the configuration of the threshold becomes intricate, leading to the risk of overlooking the arrival of the S-wave. Hence, by incorporating noise and the parameter q, the temporal fluctuation of the STA/LTA, commencing promptly after the arrival of the P-wave (more precisely, after *δ* has passed), can be assessed based on the interplay between P-waves and S-waves. This augments the reliable detection of S-waves.

**Figure 1:**
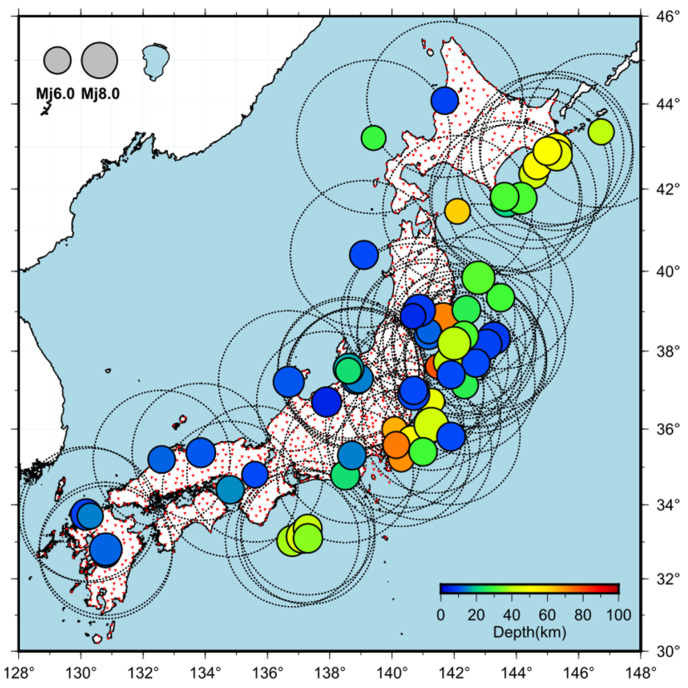
The hypocenter locations of the 65 earthquakes analyzed in this study (colored circles), the locations of K-NET stations (reverse red triangles), and the 200 km range from the epicenter of each earthquake (dash circle lines) are shown.

In this study, we selected earthquakes of magnitude ≥ M5.5 and depths ≤ 100 km, which occurred between 2003 and 2018, and manually picked S-wave arrival times on the seismic waveforms recorded by K-NET (Kyoshin net: nationwide strong motion networks in Japan [23]) for the selected earthquakes (Figure 1). The waveform data used in this study were obtained from K-NET stations, located within 200 km from the epicenter of each earthquake. Only seismic events with 20 or more K-NET stations where the arrival time of the S-wave could be interpreted manually were considered. The 65 earthquakes considered in this analysis are listed in Table 1, totaling 3030 waveforms. In this study, we computed ${STA}_{p}/{LTA}_{p}$ using the vertical component of the waveform recorded by each seismometer. The parameters *q* and ${STA}_{s}/{LTA}_{s}$ were derived from the combined waveform, obtained through vector synthesis of the two horizontal components recorded by each seismometer. The propagation speed of seismic waves is generally slowest near the Earth’s surface and increases with depth. Consequently, the propagation path of seismic waves forms a convex shape, causing the waves to approach the surface almost vertically. As a result, P-waves exhibit predominant vertical motion, while S-waves exhibit predominant horizontal motion. The acceleration waveforms recorded by K-NET were integrated to velocity waveforms before being used in the analysis. This was performed to augment the amplitude variation in the time domain and aid in the detection of S-waves. The bandwidth range of the dataset used in this study is 0.1–20 Hz.

## 3. Results

**Figure 2:**
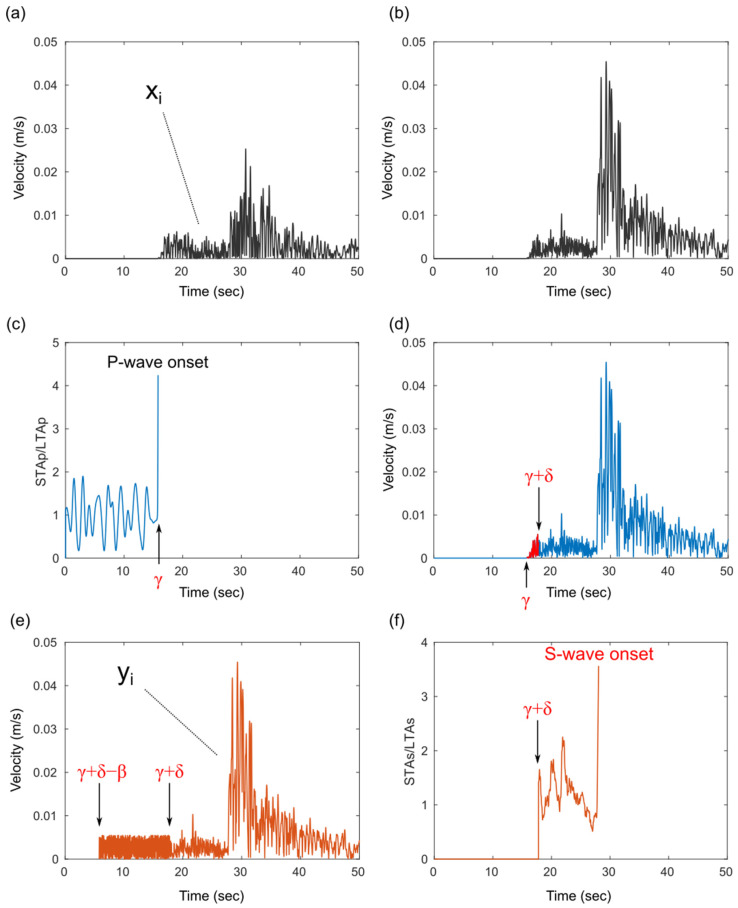
The Two-Step STA/LTA method. (**a**) The absolute vertical component waveform. (**b**) Combined horizontal component waveform. (**c**) The ${STA}_{p}/{LTA}_{p}$ calculated for the vertical component waveform ($x_{i}$) depicted in (**a**). (**d**) The segment where q is calculated for the combined horizontal two-component waveform. (**e**) Waveform $y_{i}$ replaced by *q* from *γ* + *δ* − *β* to *γ* + *δ*. (**f**) The ${STA}_{s}/{LTA}_{s}$ calculated for $y_{i}$ depicted in (**e**).

Figure 2 shows the application of the proposed method to a specific seismic waveform recorded at a given K-NET station. The absolute value of the vertical component recorded by each seismometer is shown in Figure 2a, while Figure 2b presents the absolute value of the composite waveform obtained by vector synthesis of the two horizontal velocity components recorded by each seismometer. Figure 2c illustrates the calculated ${STA}_{p}/{LTA}_{p}$, which determines the arrival time of the P-wave (*γ*) when the ${th}_{p}$ (4.5 in this case) is exceeded. The red waveforms in Figure 2d indicate the composite waveform, which is used to calculate the percentile parameter *q*. Figure 2e shows the waveform $y_{i}$, in which *q* × *N* is substituted from *γ* + *δ* − *β* to *γ* + *δ*. Finally, Figure 2f shows the ${STA}_{s}/{LTA}_{s}$ which determines the arrival time of the S-wave when the ${th}_{s}$ (3.5 in this case) is exceeded. A comparison of Figure 2a,c, which are aligned on the same temporal axis, shows that the ${STA}_{p}/{LTA}_{p}$ surpasses the ${th}_{p}$ precisely at the moment of the P-wave’s arrival. Similarly, by comparing Figure 2b,f, which are also aligned on the same temporal axis, it can be observed that the ${STA}_{s}/{LTA}_{s}$ of the proposed method surpasses the ${th}_{s}$ precisely at the moment of the S-wave’s arrival.

The proposed method was used to determine the arrival times of S-waves for the 65 earthquakes listed in Table 1. The parameters ${th}_{p}$ = 5.0, $\alpha_{p}$ = 0.5, $\beta_{p}$ = 5.0, ${th}_{s}$ = 2.2, $\alpha_{s}$ = 0.5, $\beta_{s}$ = 5.0, and *δ* = 2.0 (initial value) were utilized in this study. The parameter *β* was set to 5 s to ensure a sufficiently large value for observing the long-term background noise level, while avoiding an excessively high average value due to repeated earthquakes. The parameter *α* was set to 0.5 s to balance early detection with reducing false positives from pulse-like noise. Setting α to a shorter duration, such as 0.1 s, increases the risk of false detection from noise, while a longer duration, like 1 s, delays the detection after the P-wave arrival.

**Figure 3:**
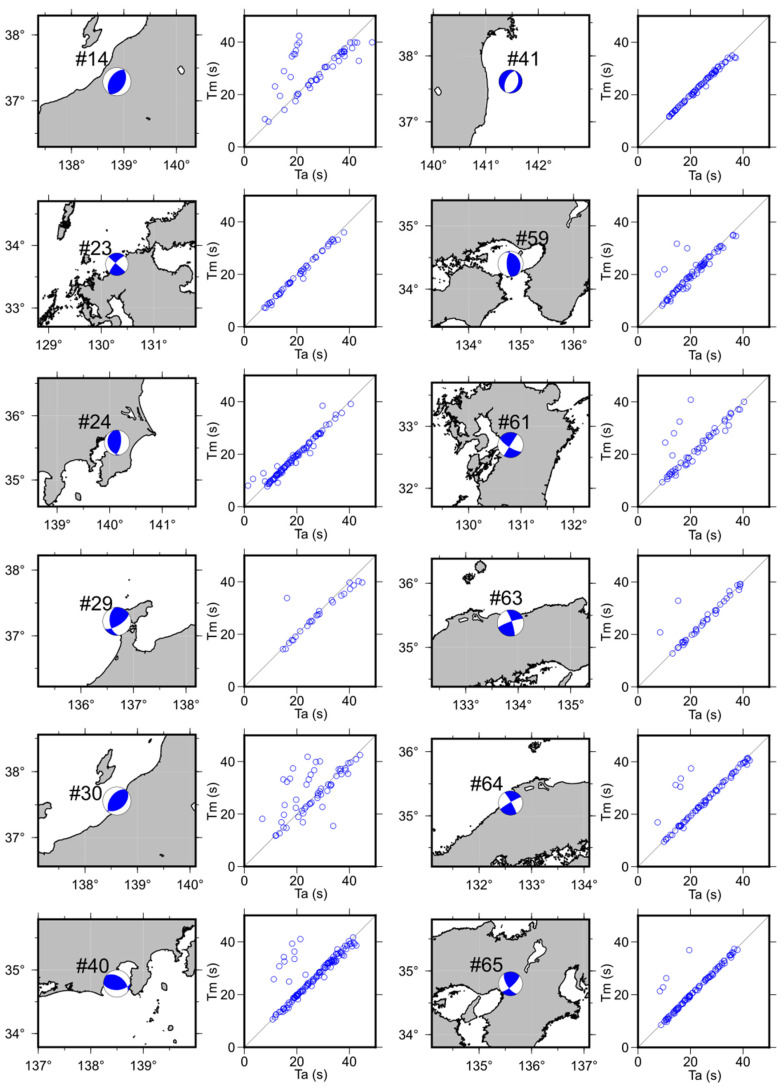
Earthquake mechanisms and comparison of S-wave detection time between automatic reading ($T_{a}$) and manual reading ($T_{m}$).

In addition, manual and automatic readings were compared for 65 earthquakes. Further, the S-wave arrival time detected by the proposed method ($T_{a}$, automatic reading) was compared with that determined through manual picking ($T_{m}$, manual reading). These are the elapsed times from the earthquake occurrence. The results of the comparison between $T_{a}$ and $T_{m}$ at each K-NET station for 12 earthquakes are shown in Figure 3. The results show that the $T_{a}$ values are accurate and aligned with the $T_{m}$ values as they are roughly aligned in a straight line. However, at certain stations, the S-wave arrival times were not accurately determined using the proposed method. This can be attributed to two primary factors that hinder the accurate automatic picking: (1) in cases where the ${STA}_{s}/{LTA}_{s}$ exceed the threshold value ${th}_{s}$ prior to the actual S-wave arrival (minus reading; $T_{a}-T_{m}<-2.0$), which is mainly evident in the comparison of $T_{a}$ and $T_{m}$ in Figure 3 for earthquakes #14, #30, and #40; (2) when the ${STA}_{s}/{LTA}_{s}$ do not exceed the designated ${th}_{s}$ value.

**Figure 4:**
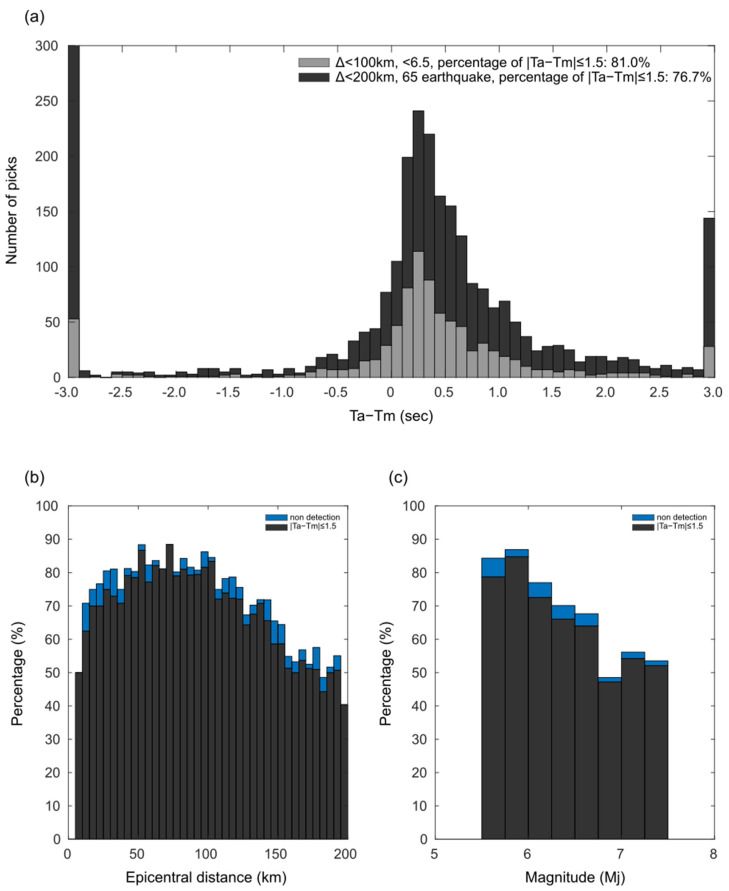
The error in automatic reading ($T_{a}$). (**a**) The histogram of residuals from waveform data obtained at stations within 200 km of the epicenter for all 65 earthquakes, totaling 3030 waveforms (black). The histogram of residuals from waveform data obtained at stations within 100 km of the epicenter for earthquake with Mj5.5–6.5, totaling 1137 waveforms (gray). (**b**) The percentage of correct decisions (${|T}_{a}-T_{m}|\leq 1.5$) vs. epicentral distance of stations (black) that do not exceed ${th}_{s}$ and cannot determine the S-wave arrival time (blue). (**c**) The percentage of correct decisions (${|T}_{a}-T_{m}|\leq 1.5$) vs. magnitude of the earthquake (black) that do not exceed ${th}_{s}$ and cannot determine the S-wave arrival time (blue).

Figure 4a shows the histograms of the residuals ($T_{a}-T_{m}$). The percentage of correct decisions ($\left|T_{a}-T_{m}\right|\leq 1.5$) for waveforms from stations located within 200 km of the epicenter for 65 earthquakes in Table 1, totaling 3030 waveforms, was approximately 76.7% (Figure 4a and Table 2). Moreover, the percentage of correct decisions decreases as the hypocenter distance increases (Figure 4b). As the hypocenter distance increases, the percentage of waveforms that do not exceed ${th}_{s}$ and thus cannot determine the S-wave arrival time also increases. The percentage of correct decisions tends to decrease slightly as the magnitude of the earthquake increases (Figure 4c). It is anticipated that the decrease in detection rate is not due to the magnitude of the earthquakes, but because larger magnitude earthquakes tend to occur in subduction zones, which are farther from the seismic observation network on land. As stated in the Introduction, when the S-wave arrival time cannot be determined, the S-wave amplitude is utilized instead of the P-wave amplitude to estimate the earthquake magnitude, and a larger magnitude than the actual one is estimated, leading to an overestimation of the warning range. This issue is particularly crucial for moderate earthquakes (~M6) because issuing warnings in unnecessary areas can have a significant social impact. Using the proposed method, an accuracy of 81.0% was achieved for waveforms from stations located within 100 km of the epicenter for earthquakes with magnitudes of 5.5–6.5 (moderate earthquakes) and depths ≤ 100 km, totaling 1137 waveforms (Figure 4a and Table 2). This result indicates that the developed method contributes to improving the accuracy of alerts.

## 4. Discussion

As with other phase detection methods, it is important to assess the accuracy of this method. The increase in the number of waveforms that do not exceed the ${th}_{s}$ level with increasing epicentral distance (Figure 4b) can be attributed to the attenuation of the amplitude in the rising portion of the P-wave, and the reduced difference between the amplitudes of the P- and S-waves due to the prevalence of reflected waves. Conversely, the phases generated by the sedimentary layers, such as PS-converted and SP-converted waves, could potentially influence the minus readings. This phenomenon is more likely to occur when a notable discontinuity surface exists directly beneath a station, such as a station situated in a basin.

**Figure 5:**
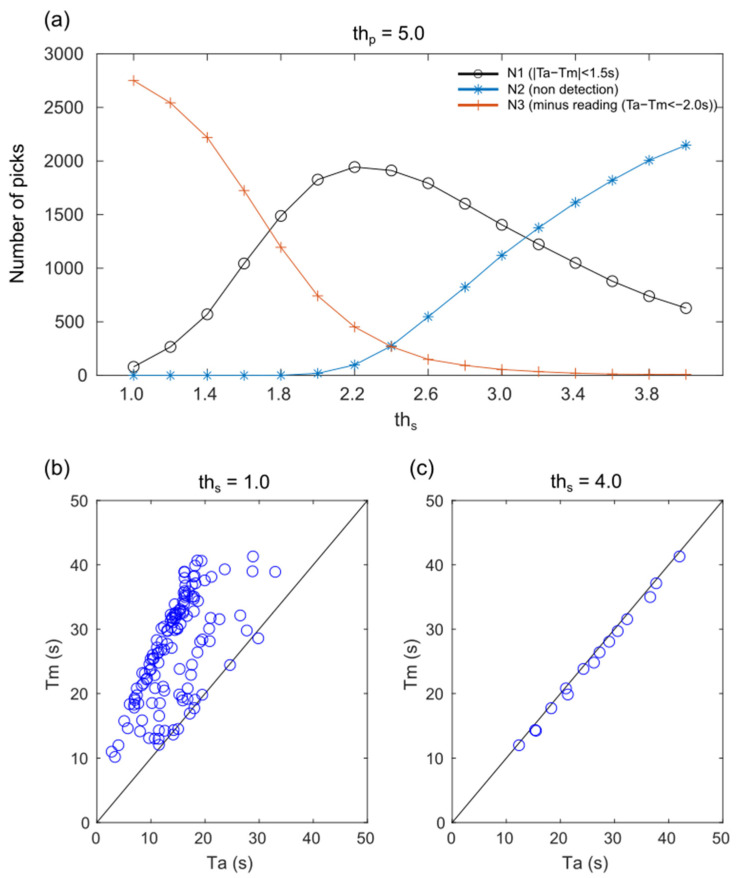
(**a**) The number of picks for N1 (black), N2 (blue), and N3 (red) were calculated by varying the threshold, ${th}_{s}$, from 1.0 to 4.0 for all stations located within an epicentral distance of less than 200 km for the 65 earthquakes. (**b**) Comparison between automatic reading ($T_{a}$) and manual reading ($T_{m}$) of earthquake #40 for ${th}_{s}$ = 1.0. (**c**) Comparison between $T_{a}$ and $T_{m}$ of earthquake #40 for ${th}_{s}$ = 4.0.

To minimize S-wave detection errors, parameter study of the ${th}_{s}$ was conducted. The ${th}_{s}$ was varied, from 1.0 to 4.0 (th_p_ fixed at 5.0), for stations within epicenter distance of less than 200 km for the 65 earthquakes. Among the picks computed using the Two-Step STA/LTA method, *N*1 represents the count of picks with $\left|T_{a}-T_{m}\right|\leq 1.5$, *N*2 represents the count of picks where the ${STA}_{s}/{LTA}_{s}$ did not surpass ${th}_{s}$, and *N*3 represents the count of picks with $T_{a}-T_{m}\leq -2.0$ (minus reading), as shown in Figure 5a. *N*1 was the highest when ${th}_{s}$ was 2.2, and as ${th}_{s}$ increased, *N*2 increased and *N*3 decreased. The decrease in *N3* with increasing ${th}_{s}$ is due to the fact that the threshold is surpassed by the S-wave rather than the PS- or SP-converted waves. However, an elevated threshold value may lead to a delayed $T_{a}$ in comparison to $T_{m}$ because the threshold is exceeded at higher amplitudes. As an example, the results for earthquake #40 computed with ${th}_{s}$ = 1.0 and ${th}_{s}$ = 4.0 are presented in Figure 5b,c. The figures illustrate that reducing the ${th}_{s}$ increases the number of minus readings, while increasing the ${th}_{s}$ reduces the number of picks and leads to a delay in $T_{a}$. Owing to the inherent variability in the observed seismic waveforms caused by factors such as source, propagation path, and site effects, it is theoretically essential to establish a specific threshold value for each station to accurately determine $T_{a}$. It may be feasible to analyze numerous earthquakes and determine thresholds empirically based on information such as ${STA}_{p}/{LTA}_{p}$ and the P-wave amplitude.

The bandwidth of the recorded seismic trace plays an important role in the performance of a phase detector, i.e., a bandwidth that is too narrow can deteriorate the efficiency of the employed algorithm [24]. Ref. [25] indicating that a strong bandwidth dependence of the STA/LTA algorithm is expected for P-wave detection, and that the STA/LTA algorithm requires a minimum bandwidth (more than 15 Hz) to reliably estimate (less than 10 samples) the phase arrival. Here, we compared the results of the Two-Step STA/LTA analysis of the seismic waveforms using five bandpass filters (0.01–20 Hz, 0.05–20 Hz, 0.1–20 Hz, 0.5–20 Hz, and 1–20 Hz). The target earthquakes and parameters are consistent with those presented in the Section 3. The percentage of correct decisions ($\left|T_{a}-T_{m}\right|\leq 1.5,\;\leq M6.5,\;\leq 100\;km$) were 80.5%, 80.0%, 81.0%, 60.2%, and 75.3%, respectively (Figure 4a and Figure S1a–4a). The percentage increased with the inclusion of low-frequency components. Nonetheless, the optimal bandwidth configuration may vary based on factors such as the magnitude (dominant frequency) and hypocentral distance (attenuation) of the station.

Although not a real-time process, previous studies have shown that in automated S-wave picking, the percentage of correct decisions often exceeds 90% with $\left|T_{a}-T_{m}\right|\leq 0.1$ [15]. This is in contrast to the 81.0% correct decisions with $\left|T_{a}-T_{m}\right|\leq 1.5$ observed (Section 3). In machine learning and statistical applications, ensemble methods, which incorporate multiple methods, outperform single-model techniques when there is diversity among them [26]. This phenomenon was also observed in phase picking [27]. Considering the objective of real-time S-wave picking, we acknowledge the constraints on our capacity to perform intricate computations. However, we propose the integration of our methodology with existing event and phase detection techniques to enhance the accuracy of the picking process.

**Figure 6:**
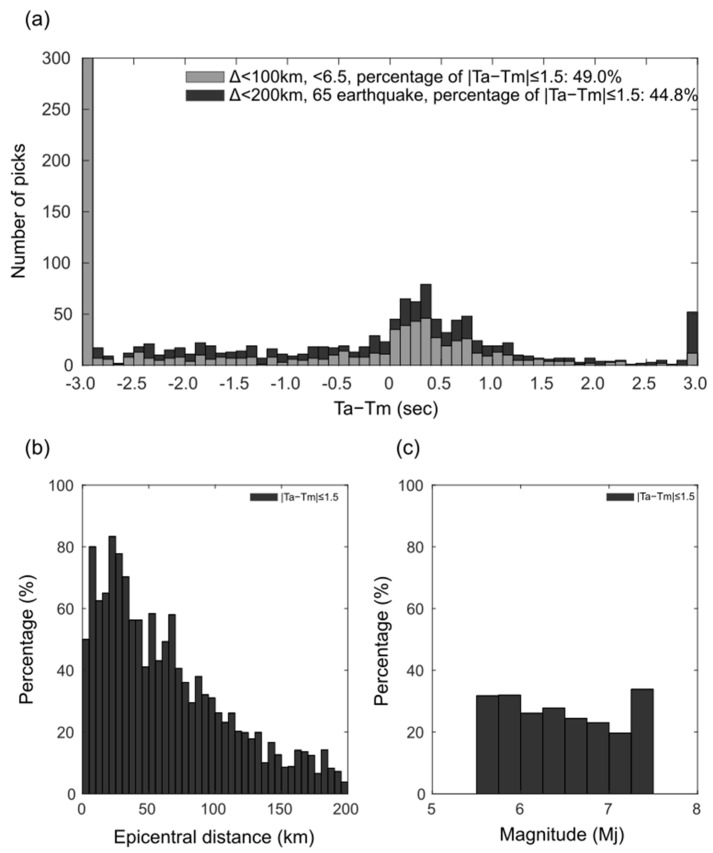
The error in automatic reading with the current method of railway EEW [1] ($T_{a}$). (**a**) The histogram of residuals from waveform data obtained at stations within 200 km of the epicenter for all 65 earthquakes (black). The histogram of residuals from waveform data obtained at stations within 100 km of the epicenter for earthquake with Mj5.5–6.5 (gray). (**b**) The percentage of correct decisions (${|T}_{a}-T_{m}|\leq 1.5$) vs. epicentral distance of stations (black). (**c**) The percentage of correct decisions (${|T}_{a}-T_{m}|\leq 1.5$) vs. magnitude of the earthquake (black).

We compare the results of our developed method with the real-time S-wave detection method currently used in railway EEW systems, which monitors the amplitude ratio of the combined horizontal two-component and vertical one-component seismic waveforms over time [1]. They stated that P-waves generally exhibit predominantly vertical motion, while S-waves exhibit predominantly horizontal motion. This difference enables the identification of P- and S-waves in the currently operational method. However, it should be noted that this distinction may not always apply to shallow or nearby earthquakes, where these characteristics can vary. The vertical and horizontal squared amplitudes are smoothed exponentially, and the amplitude ratio is calculated as follows:

(9) $$V\left(i\right)=(1-\alpha )\times X_{ud}(i)+V\left(i-1\right)\times \alpha$$

(10) $$H\left(i\right)={\left(1-\alpha \right)\times X}_{ho}(i)+H\left(i-1\right)\times \alpha$$

(11) $$HV(i)=\frac{H\left(i\right)}{V\left(i\right)}$$

where $X_{ud}(i)$ and $X_{ho}(i)$ are the absolute vertical and horizontal two-component composite waveform amplitudes, respectively. *i* is the current time step, and *α* is the exponential smoothing coefficient. An S-wave is considered to have arrived when the H/V value exceeds a set threshold after the P-wave arrival. Table 1 compares the S-wave arrival times detected using the H/V method with manually-read accurate values for all earthquakes. The proportion of correct detections within 1.5 s for all data was approximately 44.8%, and for earthquakes of magnitude 5.5–6.5 within 100 km of the epicenter, it was approximately 49.0% (Figure 6 and Table 2). It was observed that the method performs better for earthquakes with smaller epicentral distances. However, many instances detected the S-wave earlier than the actual arrival time, suggesting significant influence from PS converted waves. As mentioned earlier, the developed method shows a correct detection rate of 76.7% for 65 earthquakes within 200 km of the epicenter and over 81.0% for magnitude 5.5–6.5 earthquakes within 100 km of the epicenter (Table 2). This indicates that our proposed method can dramatically improve the accuracy of S-wave detection times while maintaining the same low computational cost as the current method.

The moderate earthquakes occur before the mainshock, leading to failures in the EEW system’s P-wave detection and magnitude estimation. In this study, we tested our developed method using files where seismic waveforms were segmented by individual earthquakes. As a result, we did not sufficiently verify the robustness of the method against successive earthquakes. Moving forward, it is essential to address successive earthquakes to enhance the accuracy of early earthquake detection for warning systems.

## 5. Conclusions

We developed a more robust method to determine the arrival time of S-waves in real time, with a low computational cost, while adhering to the basic algorithm of STA/LTA. In this approach, noise is intentionally added to the waveform ahead of the P-wave to accentuate the contrast between the P- and S-waves, followed by a renewed implementation of the STA/LTA to detect S-waves once the P-wave is successfully identified. The method can effectively determine the S-wave arrival time. The percentage of correct decisions ($\left|T_{a}-T_{m}\right|\leq 1.5$) for waveforms from stations located within 200 km of the epicenter for 65 earthquakes in Table 1 was approximately 76.7%. However, the percentage of correct answers for earthquakes of magnitude 5.5–6.5 with observation points less than 100 km was 81.0%. The error in automatic readings can be minimized by optimizing the parameters for each individual earthquake and potentially for each station. Our newly developed method for detecting S-wave arrival times in railway EEW systems has demonstrated significant improvements compared to the existing approach, which uses the amplitude ratio between horizontal and vertical seismic wave components. The current method achieves a 49.0% detection rate within 1.5 s of the true value for earthquakes of magnitude 5.5–6.5 with epicentral distances less than 100 km. In contrast, our method significantly enhances detection accuracy. Moreover, the computational cost of our method is comparable to the current method, making it straightforward to integrate into railway EEW systems. We plan to proceed with the implementation of this improved method into the system.

## Figures and Tables

**Table 1:** Earthquake list. This earthquake catalog was obtained from the Japan Meteorological Agency (JMA) website (https://www.data.jma.go.jp/eqev/data/daily_map/index.html, last accessed on 14 November 2024).

No.	Date	Origin Time	Latitude (deg)	Longitude (deg)	Depth (km)	JMA Magnitude (Mj)
#1	26 May 2003	18:24:33.4	38.8200	141.6500	72.0	7.1
#2	26 July 2003	07:13:31.5	38.4050	141.1700	12.0	6.4
#3	26 July 2003	16:56:44.5	38.5000	141.1883	12.0	5.5
#4	20 September 2003	12:54:52.2	35.2180	140.3000	70.0	5.8
#5	26 September 2003	06:08:01.8	41.7100	143.6910	21.0	7.1
#6	29 September 2003	11:36:55.0	42.3600	144.5530	43.0	6.5
#7	8 October 2003	18:06:56.7	42.5650	144.6690	51.0	6.4
#8	31 October 2003	10:06:30.6	37.8310	142.6940	33.0	6.8
#9	5 September 2004	19:07:08.0	33.0310	136.7970	38.0	6.9
#10	5 September 2004	23:57:16.8	33.1460	137.1390	44.0	7.4
#11	7 September 2004	08:29:36.2	33.3580	137.2920	41.0	6.4
#12	8 September 2004	23:58:23.1	33.1000	137.3000	36.0	6.5
#13	6 October 2004	23:40:40.1	35.9883	140.0883	66.0	5.7
#14	23 October 2004	17:56:00.3	37.2917	138.8667	13.0	6.8
#15	23 October 2004	18:11:56.7	37.2530	138.8290	12.0	6.0
#16	23 October 2004	18:34:05.6	37.3050	138.9300	14.0	6.5
#17	29 November 2004	03:32:14.5	42.9450	145.2750	48.0	7.1
#18	6 December 2004	23:15:12.0	42.8000	145.3000	46.0	6.9
#19	14 December 2004	14:56:10.5	44.0767	141.6983	9.0	6.1
#20	18 January 2005	23:09:06.6	42.9000	145.0000	50.0	6.4
#21	20 March 2005	10:53:40.3	33.7383	130.1750	9.0	7.0
#22	11 April 2005	07:22:15.6	35.7267	140.6200	52.0	6.1
#23	20 April 2005	06:11:26.8	33.7000	130.3000	14.0	5.8
#24	23 July 2005	16:34:56.3	35.5817	140.1383	73.0	6.0
#25	16 August 2005	11:46:25.7	38.1483	142.2767	42.0	7.2
#26	2 December 2005	22:13:07.9	38.0720	142.3530	40.0	6.6
#27	5 December 2005	07:20:23.0	37.8670	142.6550	25.0	5.5
#28	13 December 2005	06:01:37.5	43.2080	139.4130	29.0	5.5
#29	25 March 2007	09:41:57.9	37.2200	136.6850	11.0	6.9
#30	16 July 2007	10:13:22.5	37.5567	138.6083	17.0	6.8
#31	16 July 2007	15:37:40.4	37.5000	138.6000	23.0	5.8
#32	9 October 2007	02:10:35.4	43.3520	146.7250	40.0	5.8
#33	29 April 2008	14:26:05.3	41.4620	142.1080	62.0	5.7
#34	14 June 2008	08:43:45.3	39.0283	140.8800	8.0	7.2
#35	14 June 2008	09:20:11.8	38.8800	140.6770	6.0	5.7
#36	21 July 2008	20:30:26.6	37.1350	142.3400	27.0	6.1
#37	11 September 2008	09:20:51.3	41.7750	144.1500	31.0	7.1
#38	1 February 2009	06:51:51.8	36.7170	141.2780	47.0	5.8
#39	5 June 2009	12:30:33.8	41.8120	143.6200	31.0	6.4
#40	11 August 2009	05:07:05.7	34.7850	138.4983	23.0	6.5
#41	13 March 2010	21:49:46.8	37.6142	141.4717	77.7	5.5
#42	14 March 2010	17:08:04.1	37.7233	141.8167	40.0	6.7
#43	10 August 2010	14:50:34.6	39.3480	143.4930	30.0	6.3
#44	9 March 2011	11:45:00.0	38.3280	143.2780	8.0	7.3
#45	10 March 2011	06:23:59.7	38.1720	143.0430	9.0	6.8
#46	11 March 2011	15:06:10.7	39.0420	142.3970	27.0	6.4
#47	11 March 2011	15:09:00.0	39.8380	142.7800	32	7.4
#48	11 March 2011	15:15:00.0	36.1080	141.2650	43	7.7
#49	12 March 2011	04:46:47.6	40.4000	139.1000	10.0	6.4
#50	13 March 2011	10:26:02.0	35.8000	141.9000	10.0	6.4
#51	14 March 2011	15:12:33.9	37.7000	142.7000	10.0	6.3
#52	15 March 2011	22:31:46.3	35.3080	138.7130	14.0	6.4
#53	22 March 2011	18:19:05.2	37.4000	141.9000	10.0	6.3
#54	28 March 2011	07:23:57.0	38.3920	142.3150	31.0	6.5
#55	7 April 2011	23:32:43.4	38.2000	142.0000	40.0	7.4
#56	11 April 2011	17:16:12.0	36.9000	140.7000	10.0	7.1
#57	12 April 2011	08:08:15.8	35.4000	141.0000	30.0	6.3
#58	12 April 2011	14:07:42.2	37.0000	140.7000	10.0	6.3
#59	13 April 2013	05:33:00.0	34.4000	134.8000	15.0	6.3
#60	22 November 2014	22:08:00.0	36.7000	137.9000	5.0	6.7
#61	14 April 2016	21:26:00.0	32.7000	130.8000	11.0	6.5
#62	16 April 2016	01:25:00.0	32.8000	130.8000	12.0	7.3
#63	21 October 2016	14:07:00.0	35.3800	133.8550	11.0	6.6
#64	9 April 2018	01:32:00.0	35.2000	132.6000	12.0	6.1
#65	18 June 2018	07:58:00.0	34.8000	135.6000	10.0	5.9

**Table 2:** The comparison results of ${|T}_{a}-T_{m}|$ between our method and current method [1]. Displayed as NaN when not applicable.

	Our Method			Current Method [1]		
No.	Num. of Data |Ta − Tm|≤ 1.5 Δ ≤ 100 km	Num. of Data Δ ≤ 100 km	Correct Decision (%) Δ ≤ 100 km	Num. of Data |Ta − Tm| ≤ 1.5 Δ ≤ 100 km	Num. of Data Δ ≤ 100 km	Correct Decision (%) Δ ≤ 100 km
#1	15	18	83.33	11	18	61.11
#2	9	18	50.00	11	18	61.11
#3	20	27	74.07	10	27	37.04
#4	34	39	87.18	27	39	69.23
#5	0	4	0.00	0	4	0.00
#6	5	8	62.50	4	8	50.00
#7	3	4	75.00	1	4	25.00
#8	0	0	NaN	0	0	NaN
#9	0	0	NaN	0	0	NaN
#10	0	0	NaN	0	0	NaN
#11	0	0	NaN	0	0	NaN
#12	0	0	NaN	0	0	NaN
#13	54	69	78.26	46	69	66.67
#14	9	14	64.29	5	14	35.71
#15	18	20	90.00	12	20	60.00
#16	21	25	84.00	15	25	60.00
#17	3	3	100.00	0	3	0.00
#18	14	15	93.33	5	15	33.33
#19	10	13	76.92	8	13	61.54
#20	19	23	82.61	10	23	43.48
#21	14	20	70.00	6	20	30.00
#22	24	40	60.00	7	40	17.50
#23	35	42	83.33	18	42	42.86
#24	51	71	71.83	37	71	52.11
#25	0	0	NaN	0	0	NaN
#26	0	0	NaN	0	0	NaN
#27	0	0	NaN	0	0	NaN
#28	8	8	100.00	3	8	37.50
#29	7	9	77.78	4	9	44.44
#30	12	17	70.59	13	17	76.47
#31	28	36	77.78	21	36	58.33
#32	3	3	100.00	0	3	0.00
#33	11	11	100.00	3	11	27.27
#34	5	10	50.00	4	10	40.00
#35	24	37	64.86	11	37	29.73
#36	0	0	NaN	0	0	NaN
#37	1	2	50.00	0	2	0.00
#38	16	16	100.00	8	16	50.00
#39	1	2	50.00	0	2	0.00
#40	37	43	86.05	24	43	55.81
#41	22	22	100.00	8	22	36.36
#42	5	7	71.43	3	7	42.86
#43	0	0	NaN	0	0	NaN
#44	4	5	80.00	2	5	40.00
#45	0	0	NaN	0	0	NaN
#46	15	20	75.00	16	20	80.00
#47	4	5	80.00	2	5	40.00
#48	15	20	75.00	16	20	80.00
#49	3	5	60.00	2	5	40.00
#50	1	1	100.00	1	1	100.00
#51	0	0	NaN	0	0	NaN
#52	16	20	80.00	16	20	80.00
#53	5	5	100.00	4	5	80.00
#54	3	3	100.00	1	3	33.33
#55	5	5	100.00	5	5	100.00
#56	8	12	66.67	2	12	16.67
#57	8	10	80.00	8	10	80.00
#58	38	44	86.36	24	44	54.55
#59	42	57	73.68	20	57	35.09
#60	9	21	42.86	6	21	28.57
#61	43	58	74.14	28	58	48.28
#62	5	11	45.45	3	11	27.27
#63	18	23	78.26	6	23	26.09
#64	31	36	86.11	15	36	41.67
#65	75	80	93.75	30	80	37.50
	AVG, (65 eqarthquakes)		76.65	AVG. (65 earthquakes)		44.80
	AVG. (Mj5.6–6.5)		81.04	AVG. (Mj5.6–6.5)		48.97

## Data Availability

The waveform data used in this study were obtained from the National Research Institute for Earth Science and Disaster Resilience [28]. The seismic data used in this study were obtained from the Japan Meteorological Agency [JMA, https://www.data.jma.go.jp/eqev/data/daily_map/index.html]. The Supplemental Material for this article includes Figures S1–S4 with analysis details. Part of this research has already been published as a pre-print, accessed on 14 November 2024 [29].

## Supplementary Materials

The following supporting information can be downloaded at: https://www.mdpi.com/article/10.3390/s24237452/s1, Figure S1: The error of T_a, obtained from the waveform filtered within the frequency range of 0.01–20 Hz, is presented, Figure S2: The error of T_a, obtained from the waveform filtered within the frequency range of 0.05–20 Hz, is presented, Figure S3: The error of T_a, obtained from the waveform filtered within the frequency range of 0.5–20 Hz, is presented and Figure S4: The error of T_a, obtained from the waveform filtered within the frequency range of 1.0–20 Hz, is presented,
