# Supplementary material for: Enhancing Railway Earthquake Early Warning Systems with a Low Computational Cost STA/LTA-Based S-Wave Detection Method
Source: Sensors (Basel). 2024 Nov 22;24(23):7452. doi: 10.3390/s24237452 (PMC11644365; doi:10.3390/s24237452)
Supplement: Supplementary file 1 [file sensors-24-07452-s001.zip › sensors-3265667-supplementary.pdf]

sensors

Communication

Enhancing Railway Earthquake Early Warning Systems with a Low Computational Cost STA/LTA-Based S-Wave Detection Method

Satoshi Katakami [1,*] and Naoyasu Iwata [1]

1 Supplementary Figures and Tables

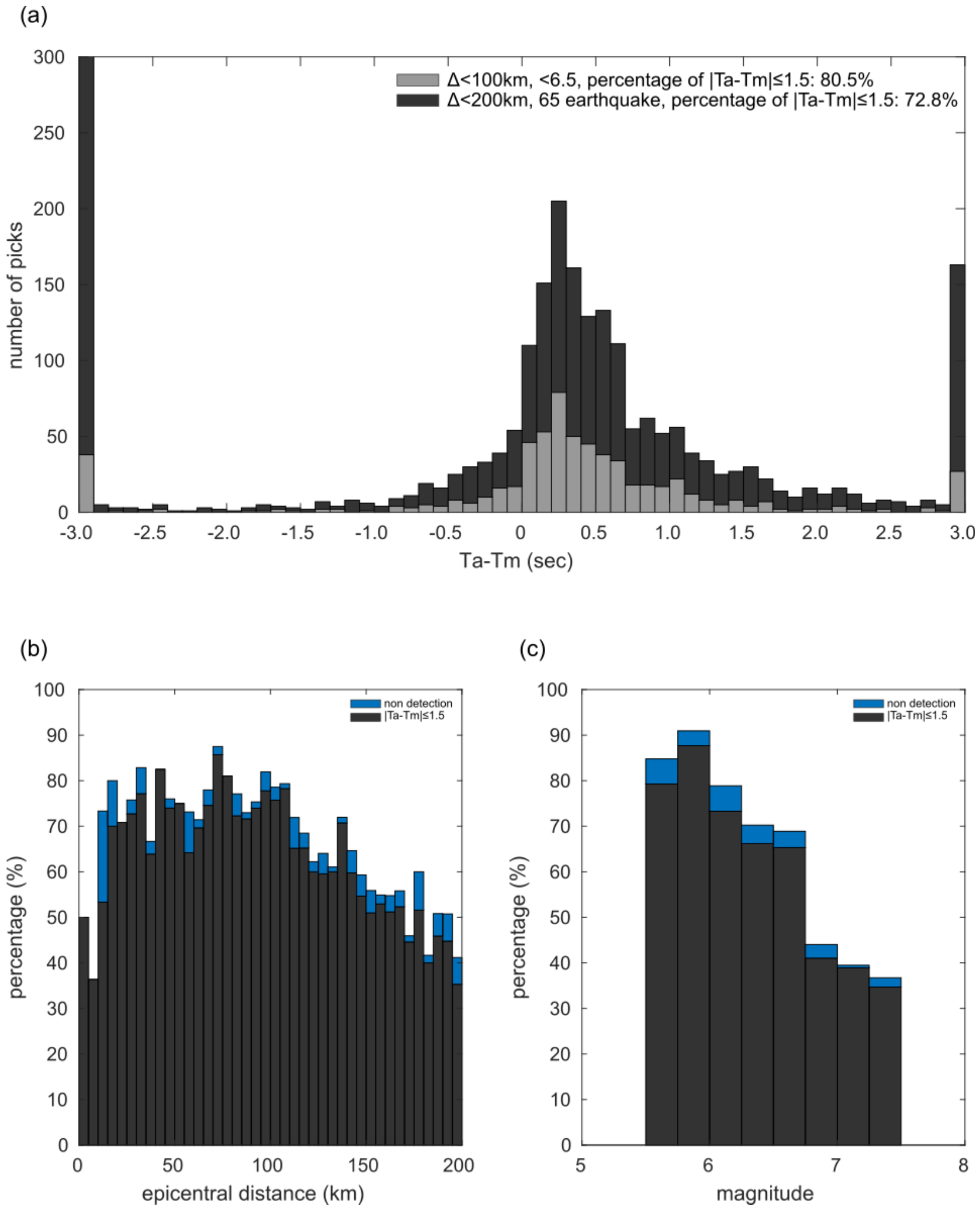

Figure S1. The error of T_a, obtained from the waveform filtered within the frequency range of 0.01-20Hz, is presented. The remaining captions are consistent with those provided in Figure 3.

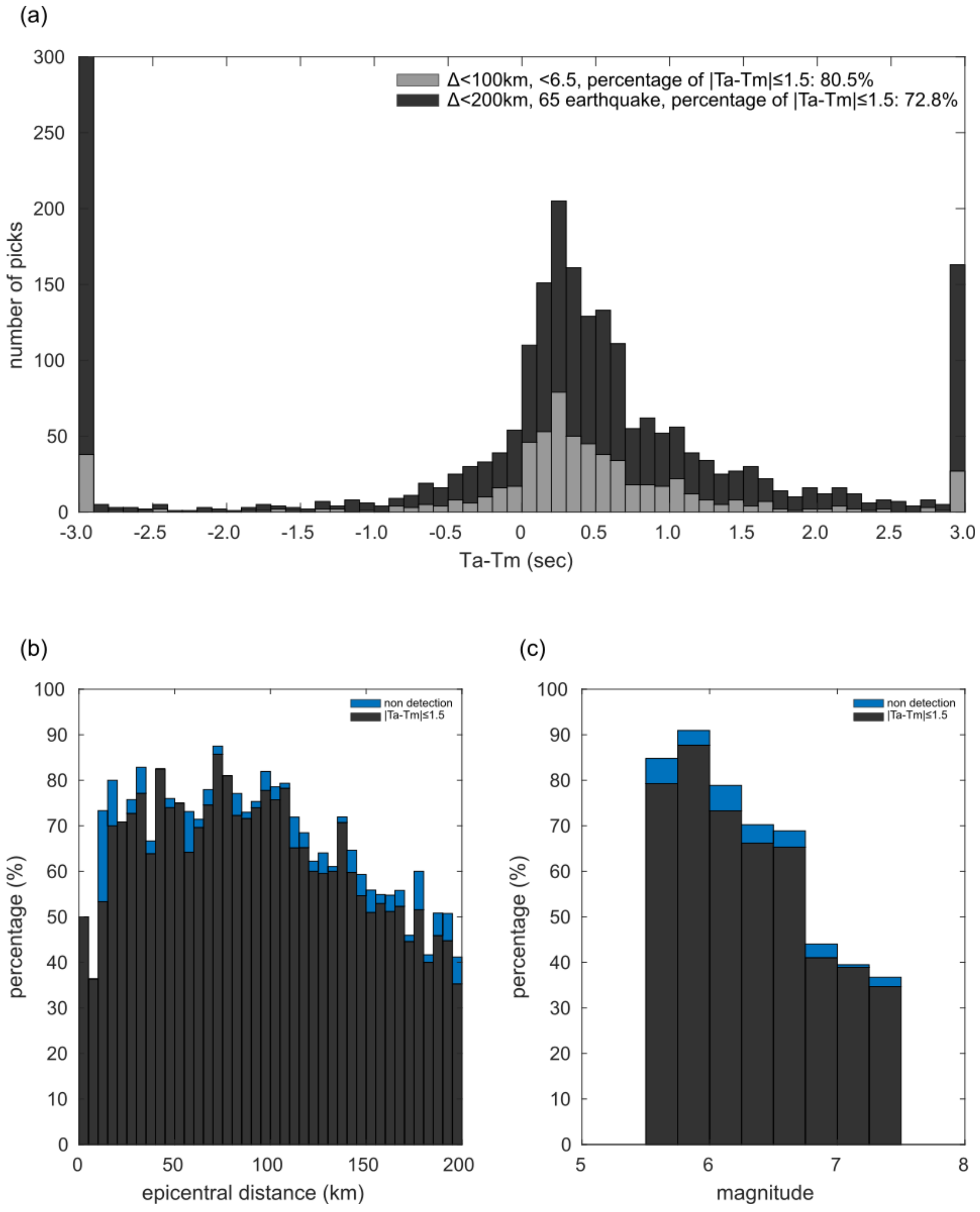

Figure S2. The error of T_a, obtained from the waveform filtered within the frequency range of 0.05-20Hz, is presented. The remaining captions are consistent with those provided in Figure 3.

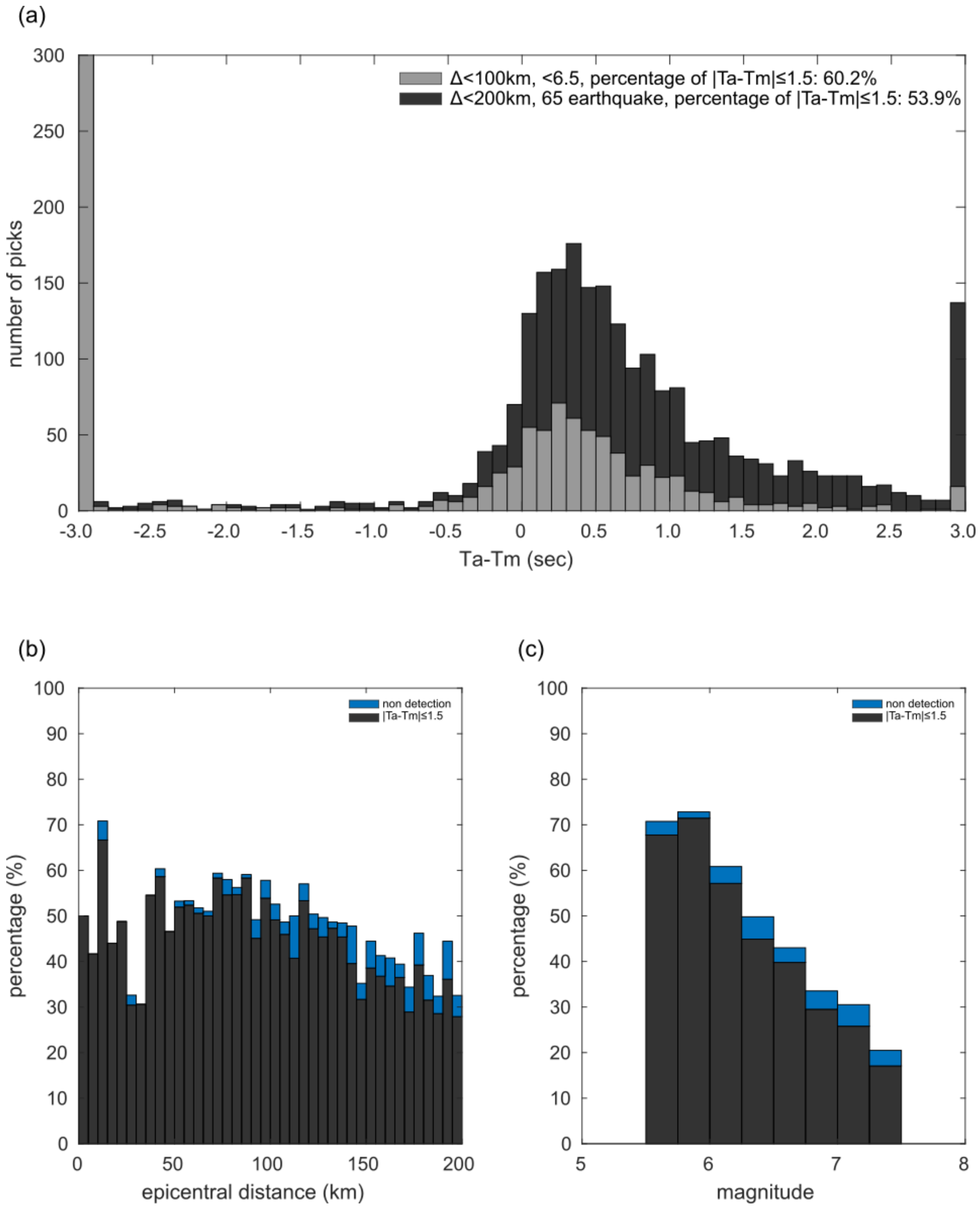

Figure S3. The error of T_a, obtained from the waveform filtered within the frequency range of 0.5-20Hz, is presented. The remaining captions are consistent with those provided in Figure 3.

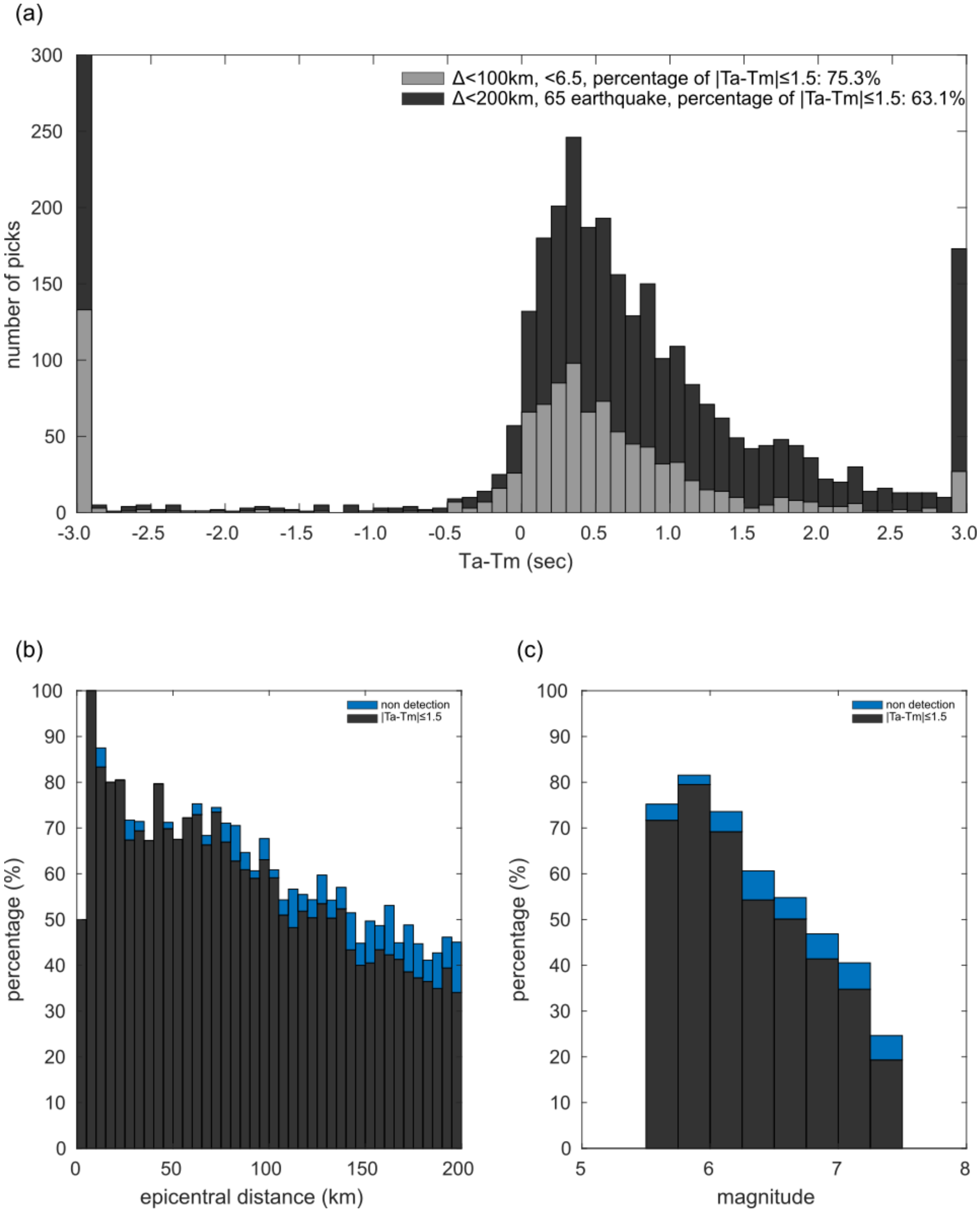

Figure S4. The error of T_a, obtained from the waveform filtered within the frequency range of 1.0-20Hz, is presented. The remaining captions are consistent with those provided in Figure 3.
